# Dynamically reprogrammable nonlinear Pancharatnam–Berry phase via ferroelectric nematic liquid crystals: a new paradigm for reconfigurable nonlinear optics

**DOI:** 10.1038/s41377-025-02086-4

**Published:** 2026-01-02

**Authors:** Shuang Zhang

**Affiliations:** 1 Department of Physics, University of Hong Kong, Hong Kong, China; 2 Department of Electrical and Electronic Engineering, University of Hong Kong, Hong Kong, China

**Keywords:** Nonlinear optics, Liquid crystals

## Abstract

A dynamically programmable, nonlinear beam-shaping and steering system is demonstrated, based on photopatterned, electrically controlled, ion-doped liquid ferroelectrics. This innovative approach elevates the linear liquid-crystal Pancharatnam–Berry optics to the reconfigurable nonlinear Pancharatnam–Berry optics regime, creating new possibilities for dynamic light-matter interactions, multiplexing holography, tunable quantum optics, and many other reconfigurable photonic applications.

The Pancharatnam–Berry (PB) phase, a type of geometric phase resulting from the adiabatic evolution of light in anisotropic media, has been extensively studied over recent decades. With the rapid advancement of nonlinear materials, the concept of PB has been extended from linear optics into the nonlinear regime, attracting significant interest for its potential for manipulating nonlinear optical processes. Nonlinear PB phases not only serve as a fundamental platform for exploring light-matter interactions in nonlinear systems but also facilitate the design of versatile optical components for applications such as wavefront shaping, imaging, and holography. Of particular interest is the prospect of tunable nonlinear materials, which could offer a reconfigurable platform for investigating dynamic light-matter interactions and creating reprogrammable, multifunctional nonlinear optical devices.

In a recent study published in *Light: Science & Applications*^1^, the team from Nanjing University has shifted their focus from solid-state ferroelectrics to liquid ferroelectrics, namely ferroelectric nematic liquid crystal (FNLC), demonstrating electrically reconfigurable nonlinear PB optics for advanced dynamic nonlinear beam shaping and steering. Liquid ferroelectrics were first theoretically predicted by Nobel laureate Max Born in 1916^2^, yet the experimental verification endured a protracted journey. It was not until 2017 that two independent research teams—the Mandle-Goodby group in the UK and the Kikuchi group in Japan—each discovered a class of strong ferroelectric liquid crystalline materials^3,4^. These materials were experimentally validated in 2020 by the research team led by Prof. N. A. Clark^5^, a member of the U.S. National Academy of Sciences. This groundbreaking discovery, owing to its profound significance in soft matter science, was named one of the Top 10 Breakthroughs of 2020 by *Physics World*, the journal of the Institute of Physics (UK). This transformative soft-matter material, characterized by its ferroelectric stacking order and macroscopic polarization characteristics, has since unveiled a multitude of unprecedented physical properties and external field responses^6–11^. Its unique attributes have also positioned it as a highly promising platform for next-generation optoelectronic devices, demonstrating immense potential in applications ranging from dynamic optical information processing to advancing nonlinear photonic technologies (Fig. 1).

**Fig. 1:**
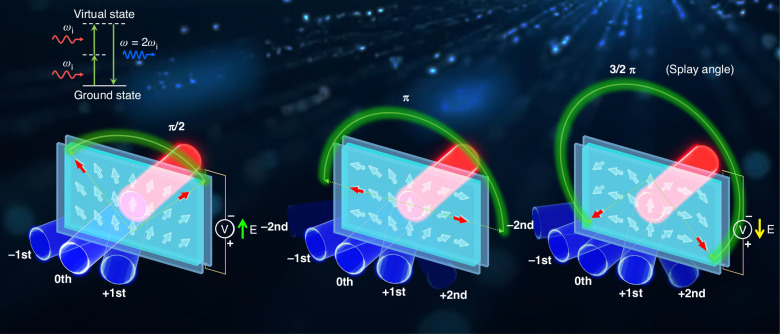
Schematic illustrations of the reconfigurable nonlinear PB diffractive optics with photopatterned ferroelectric nematics

## Bridging the nonlinear PB phase with FNLCs marks a major advancement in the field of soft-matter nonlinear photonics, enabling unprecedented control over light fields

The framework proposed in *Light: Science & Applications*^1^ features a photopatterned FNLC film with a flexoelectric-assisted^12^ periodic splay distribution of polar LC directors in the *x*-*y* plane for reconfigurable nonlinear optics. The thickness of the LC film typically ranges on the order of micrometers. Each pixel within this structure possesses a specific polar LC director orientation, which critically influences the optical response when illuminated by circularly polarized (CP) pumping light propagating along the *z*-axis. The interaction between the CP fundamental wave (FW) and the structured FNLC medium induces spin-dependent PB phase manipulation. The orientation angle *α* of the LC director relative to the laboratory’s coordinate system, along with the spin state *σ* (*σ* = ±1 for right-handed and left-handed circular polarizations), governs both linear and nonlinear PB phases.

In the linear optics regime, a CP incident wave undergoes polarization state evolution as it propagates through the LC medium. The cross-CP component of the FW acquires a spin state transformation, leading to a closed trajectory on the Poincaré sphere with a resulting PB phase shift of 2*σα*^13,14^. The nonlinear optics regime involves simultaneous changes in both polarization state and light frequency^15,16^. The interaction between the FW and LCs results in frequency conversion to the *n*th harmonic (e.g., second-harmonic generation (SHG) for *n* = 2), which amplifies the angular displacement by a factor of *n*, yielding distinct nonlinear PB phases for co- and cross-CP components of the SH wave. Specifically, the co-CP component acquires a phase shift of *σα*, while the cross-CP component experiences a phase shift of 3*σα*.

## The versatility of this system is significantly augmented by the three-dimensional engineering of polar LC directors and the capability to dynamically modulate their orientation via external electric fields

On the one hand, SHG can occur at any arbitrary depth $z={\rm{N}}\,\cdot \,{\rm{d}}z$ within the polar LC film (the film can be divided into numerous infinitesimally thin layers along its thickness direction, each of thickness d*z*). Both prior to and following the nonlinear frequency conversion event within each layer, the propagating optical fields undergo linear PB phase accumulations during their propagation through the LC medium. This sequential process—linear PB phase modulation preceding SHG, nonlinear frequency conversion, and subsequent linear PB phase modulation of the generated harmonic wave—enables the collective superposition of geometrically phased contributions from all layers, ultimately defining the total nonlinear optical response at the exit plane.

On the other hand, the dynamic control of FNLCs enables the real-time reconfiguration of the nonlinear PB phase, facilitating the dynamic steering and shaping of optical signals. As a proof of concept, the researchers fabricated a photopatterned splay-aligned FNLC grating to demonstrate active nonlinear Raman-Nath diffractive beam steering. Upon application of a low-frequency (1 Hz) in-plane electric field (±0.06 V·μm⁻¹), the electrical reorientation of polar LC directors induces transitions in the grating’s splay angle from its equilibrium state (*θ* = π) to obtuse or acute configurations depending on the field polarity. This reorientation simultaneously modulates the local orientation angle (*α*) of FNLCs. Consequently, the nonlinear PB phase undergoes real-time adjustment, allowing for the dynamic manipulation of SHG signals. Rationally designed structured electrodes could be further incorporated to enable pixelated in-plane switching of FNLCs and subsequently reprogram more optical functionalities. The ion-doping material strategy ensures smooth and stable electrical modulation, thereby enabling precise control over the optical responses.

This work demonstrates the exceptional potential of emergent FNLCs for active nonlinear photonic manipulation, offering promising applications in sophisticated optical processing, advanced imaging, and next-generation quantum information technologies. By combining inherent advantages—including tunability, programmable reconfigurability, multifunctionality, and facile fabrication—this system establishes a compelling material platform for investigating dynamic light-matter interactions. Furthermore, it significantly broadens the scope of LC technologies in nonlinear optics.
